# Mineralocorticoid Receptor Antagonists in Diabetic Kidney Disease

**DOI:** 10.3390/ph14060561

**Published:** 2021-06-11

**Authors:** Nina Vodošek Hojs, Sebastjan Bevc, Robert Ekart, Nejc Piko, Tadej Petreski, Radovan Hojs

**Affiliations:** 1 Department of Nephrology, Clinic for Internal Medicine, University Medical Centre Maribor, Ljubljanska ulica 5, 2000 Maribor, Slovenia; sebastjan.bevc@gmail.com (S.B.); tadej.petreski@gmail.com (T.P.); radovan.hojs@guest.arnes.si (R.H.); 2 Faculty of Medicine, University of Maribor, Taborska ulica 8, 2000 Maribor, Slovenia; robert.ekart2@guest.arnes.si; 3 Department of Dialysis, Clinic for Internal Medicine, University Medical Centre Maribor, Ljubljanska ulica 5, 2000 Maribor, Slovenia; nejc.piko@gmail.com

**Keywords:** diabetic kidney disease, chronic kidney disease, mineralocorticoid receptor antagonists, spironolactone, eplerenone, apararenone, esaxerenone, finerenone

## Abstract

Diabetes mellitus is a global health issue and main cause of chronic kidney disease. Both diseases are also linked through high cardiovascular morbidity and mortality. Diabetic kidney disease (DKD) is present in up to 40% of diabetic patients; therefore, prevention and treatment of DKD are of utmost importance. Much research has been dedicated to the optimization of DKD treatment. In the last few years, mineralocorticoid receptor antagonists (MRA) have experienced a renaissance in this field with the development of non-steroidal MRA. Steroidal MRA have known cardiorenal benefits, but their use is limited by side effects, especially hyperkalemia. Non-steroidal MRA still block the damaging effects of mineralocorticoid receptor overactivation (extracellular fluid volume expansion, inflammation, fibrosis), but with fewer side effects (hormonal, hyperkalemia) than steroidal MRA. This review article summarizes the current knowledge and newer research conducted on MRA in DKD.

## 1. Introduction

Diabetes mellitus (DM) is a worldwide health issue and it is estimated that it affected 463 million adults in 2019 [1]. Up to forty percent of these patients have diabetic kidney disease (DKD), making it the most common cause of chronic kidney disease [2]. DM patients suffer from excess morbidity, cardiovascular and all-cause mortality [3,4]. Cardiovascular and all-cause mortality is further increased by the presence of chronic kidney disease [5,6,7]. Patients with DM have a 10-year life span loss while those with DKD have a 16-year life span loss [8]. DM and its complications present not only a personal, but also a major economic burden [9,10].

DKD is a microvascular complication. Although the gold standard for DKD confirmation is kidney biopsy, it is mostly not performed because of its invasive nature. Clinical identification of DKD is based upon at least 3 months of decreased estimated glomerular filtration rate (eGFR < 60 mL/min/1.73 m^2^) and/or presence of albuminuria (urinary albumin–creatinine ratio (UACR) ≥ 30 mg/g) in a patient with DM. Albuminuria is also an important risk marker; it is associated with kidney disease progression, risk of end-stage kidney disease, cardiovascular morbidity and mortality [11,12]. Risk factors for DKD are various (sex, age, ethnicity, hyperglycemia, arterial hypertension, obesity, smoking, etc.), but the leading ones are hyperglycemia and arterial hypertension [13]. Therefore, treatment of DKD encompasses appropriate lifestyle changes (i.e., weight reduction, physical activity, appropriate diet, smoking cessation), glycemic and blood pressure control [14]. The cornerstone of therapy in patients with albuminuria are angiotensin-converting enzyme (ACE) inhibitors or angiotensin receptor blockers (ARB), which slow down the rate of kidney function decline [15]. However, they do not reduce the number of cardiovascular events [16]. In the last few years, sodium-glucose co-transporter-2 (SGLT2) inhibitors made it to the forefront by lowering blood sugar, blood pressure, reducing kidney function decline and improving cardiovascular outcomes [17,18,19,20]. Since 2019, SGLT2 inhibitors have been part of the recommended treatment of DKD by the American Diabetes Association. Glucagon-like peptide-1 (GLP1) agonists also improve glycemic control, decrease cardiovascular outcomes and moderately decrease kidney outcomes [21]. Other hypoglycemic agents with potential benefit in DKD are dipeptidyl peptidase 4 (DPP4) inhibitors, but further research needs to be conducted [22,23]. Despite all these treatment options, current management of DKD still leaves a substantial residual risk for kidney disease progression, morbidity and mortality. Therefore, novel treatments are being developed that target inflammation, fibrosis, oxidative stress, renal hemodynamics, glomerular hyperfiltration, the endothelin system, janus kinase (JAK)-signal transducer and activator of transcription (STAT) pathway, etc. [14,24]. Recently, a selective endothelin A receptor antagonist atrasentan was shown to lower the risk of renal events in carefully selected patients with DKD [25]. Baricitinib, a JAK1 and 2 inhibitor, was also shown to reduce albuminuria in DKD patients [26]. New potential treatment targets are continuously being recognized, such as N-acetyl-seryl-aspartyl-lysyl-proline (AcSDKP), sirtuin 3 (SIRT3), glycolysis inhibitors, pyruvate kinase M2 type (PKM2) activators, etc. [27,28]. New insights into the pathophysiology of DKD are also being obtained. For example, it was found that loss of endothelial glucocorticoid receptors accelerates diabetic nephropathy in mice [29]. However, further research and large-scale randomized controlled trials are necessary before new treatments are utilized in everyday clinical practice.

Among novel treatments, mineralocorticoid receptor antagonists (MRA) are in the limelight. The focus of this article is to conduct an in-depth review of mineralocorticoid receptors (MR) and their antagonists, focusing on the ones used in clinical practice or latest clinical research. For each of them, we make a short pharmacological introduction, followed by some key clinical trials in cardiovascular medicine because of the importance of cardiovascular diseases in DKD. This is followed by research conducted on DKD.

## 2. Mineralocorticoids and the Mineralocorticoid Receptors (MR)

The last few decades have seen a major shift in the understanding of the role of mineralocorticoids and MR at the physiological and pathophysiological level.

Mineralocorticoids are steroid hormones; the main physiological mineralocorticoid is aldosterone. It is synthesized in the outer layer of the adrenal gland in response to hyponatremia and hyperkalemia, through the activation of the renin–angiotensin system (RAS) [30]. However, it can also be produced locally in peripheral tissues [31]. Aldosterone’s classical, renal/epithelial and main role is in the control of blood pressure and extracellular volume homeostasis through sodium reabsorption and potassium excretion. Later it became clear that it also has extrarenal/non-epithelial effects by stimulating inflammation, fibrosis and necrosis. An important contribution to this knowledge came from Hans Seyle and his colleagues. They showed the pro-inflammatory and pro-fibrotic effects of mineralocorticoids and attenuation of these effects by an aldosterone antagonist spironolactone in animal models [32,33,34]. Sadly, these results were not adequately noticed and were forgotten for many decades. Awareness was raised again in the 1990s through the experiments performed by Brilla and colleagues. They showed that mineralocorticoid excess in rats contributed to cardiac fibrosis independent of high blood pressure [35,36]. Additionally, they found that the aldosterone antagonist spironolactone prevented perivascular and interstitial cardiac fibrosis in rats regardless of arterial hypertension and left ventricle hypertrophy [37].

MR are intracellular receptors and act as nuclear transcription factors (“genomic” effect) or have rapid non-genomic effects through secondary cell signaling mechanisms [38,39,40]. They are expressed in many tissues, including the kidney, heart, vasculature, nervous and immune system. Although it was originally thought that only mineralocorticoids bind to them, it was later found that aldosterone, cortisol, and progesterone bind to them with the same affinity [41]. Progesterone acts as an MR antagonist. Levels of plasma cortisol are much higher than aldosterone levels. In “aldosterone target cells” (renal tubular epithelial cells, sweat glands, salivary glands, colon, vascular endothelial cells, smooth muscle cells, some brain cells), cortisol is mostly converted by 11β-hydroxysteroid dehydrogenase type 2 (11β-HSD2) into an inactive metabolite [42,43]. Therefore, aldosterone can activate the MR even at lower concentrations. In 11β-HSD2-deficient cells, cortisol normally acts as an antagonist—it binds to the MR and does not activate it [41]. However, in tissue damage the cortisol–MR complex may become activated and, in this case, cortisol acts as an MR agonist. Cortisol may also act as an MR agonist in certain other states, as is the case in aldosterone deficiency [44]. Importantly, the aldosterone–MR complex is more stable, being 200 times more active for transactivation than the cortisol–MR complex [45]. The process of MR activation is also regulated by different cofactors in a ligand and cell-type specific way, leading to different physiological responses [46,47]. Additionally, MR expression can be modified in disease states [45]. MR affect fluid, electrolyte and hemodynamic homeostasis, as well as tissue remodeling [46]. Increased MR activity can be driven by increased levels of aldosterone, a shift in cortisol activity from MR antagonist to MR agonist or raised local expression of the MR [48]. MR overactivation can lead to inflammation, increased oxidative stress, necrosis, fibrosis and finally, organ damage. It has been linked to arterial hypertension, coronary artery disease, heart failure, metabolic syndrome, DM and chronic kidney disease [15,49,50,51,52].

## 3. Mineralocorticoid Receptor Antagonists (MRA)

Historically, MRA started as aldosterone antagonists. At that time, it was not yet known that the aldosterone receptor is promiscuous. Interestingly, the first MRA spironolactone was developed in 1957, shortly after aldosterone purification, and 30 years before the complementary DNA (cDNA) of the MRA was cloned [53]. Its structure was based on the naturally occurring antagonist progesterone. Spironolactone is a steroidal MRA and the first “anti-hormone” used in clinical practice [54]. Because of its low selectivity, newer and more selective MRA were sought, and another steroidal MRA, eplerenone, was developed. Steroidal MRA were discovered and characterized by experiments on animals and humans. In search of new, even more selective and potent MRA, pharmaceutical companies used computer screening of different compounds [53]. This was possible only after the cDNA of the whole steroid hormone receptor family was cloned. The search led to the discovery of non-steroidal MRA; currently, the most advanced compounds are apararenone, esaxerenone and finerenone. They differ from steroidal MRA in their pharmacokinetics, tissue distribution, mode of action, effects on cofactor recruitment and effects on inflammation and fibrosis [30].

MRA were first used in clinical trials in cardiovascular diseases, especially in patients with heart failure. Later, their role in chronic kidney disease evolved. The first meta-analysis looking at the role of spironolactone and eplerenone in chronic kidney disease patients already treated with RAS inhibitors showed that MRA reduce proteinuria and increase the risk of hyperkalemia [55]. In the latest meta-analysis, the use of MRA (spironolactone, eplerenone, finerenone) alone or on top of RAS inhibitors had a significant antiproteinuric effect in chronic kidney disease patients [56]. Compared to placebo, MRA decreased UACR by 24.6%, urine protein–creatinine ratio by 53.9% and 24 h albuminuria by 32.5%. All this is likely to be clinically relevant since >30% albuminuria reduction in comparison to placebo has been shown to reduce the risk of end-stage kidney disease development [57]. In the same meta-analysis, they also showed that MRA were associated with a slight decrease in eGFR (2.4 mL/min/1.73 m^2^), a small increase in mean potassium level (0.2 mEq/L) and a 2.6-fold increase in hyperkalemia risk compared with placebo/active control [56].

DKD is a form of chronic kidney disease that is mostly driven by renal and vascular inflammation, matrix formation and fibrosis [15]. Inflammation affecting the podocytes leads to albuminuria, which leads to kidney disease progression and is associated with high cardiovascular risk [58]. The inflammatory and proliferative response in DKD is stimulated by relative aldosterone excess [15]. This persists even after the use of ACE inhibitors or ARB, due to the so-called aldosterone escape phenomenon [15]. Namely, aldosterone secretion is not only stimulated by angiotensin II, but also by other stimuli like hyperkalemia and atrial natriuretic peptide [15]. MRA present a hot research topic in DKD.

## 4. Steroidal MRA

### 4.1. Spironolactone

Spironolactone is one of the spirolactones, i.e., steroids that contain a γ-lactone or a γ-hydroxy acid function at C-17 [53]. Two other important spirolactones, SC-5233 (3-(3-oxo-17β-hydroxy-4-androsten-17α-yl)propionic acid γ-lactone) and SC-8109 (19-nor analog of SC-5233) were the first studied steroidal MRA. Liddle was the first to use a steroidal MRA in humans and showed that SC-5233 induced natriuresis in seven edematous patients due to congestive heart failure or nephrosis [59]. The problem with these spirolactones was that they had to be administered parenterally to achieve a sufficient effect. Therefore, these compounds were chemically modified and in 1957 the first oral MRA spironolactone (SC-9420; 7α-acetylthio-17α-hydroxy-3-oxopregn-4-ene-21-carboxylic acid γ-lactone) was developed [53]. In 1960 it was approved by the FDA for use as a diuretic in the management of edema, arterial hypertension and primary hyperaldosteronism.

Spironolactone binds to the MR at the same site as aldosterone. It has a high affinity for the MR, but dissociates more quickly, destabilizes the receptor and hinders coactivator recruitment [60]. Depending on promoters and cell type, it also has partial agonist effects [60]. It is highly bound to plasma proteins (around 90%). It has a short half-life of 1–2 h, and is metabolized into more active metabolites, the most important ones being 7α-thiomethylspironolactone and canrenone [42,61]. In some countries, canrenone is also marketed as an independent drug formulation. The main metabolites have half-lives of 18–24 h; therefore, spironolactone can be administered once daily [42]. Based on quantitative whole-body autoradiography in rodents, it reaches a six times higher concentration in the kidneys than in the heart [60]. Spironolactone is not a very selective MR antagonist, it also acts as an antagonist of the androgen receptor leading to gynecomastia and impotence, and as an agonist of the progesterone receptor causing menstrual irregularities [42]. Another important side effect, especially in chronic kidney disease patients receiving RAS inhibitors, is hyperkalemia. Despite its side effects, it is still in widespread clinical use. It is prescribed in doses from 12.5 mg and up to 400 mg daily, depending on the indication [62].

The first important clinical trials with spironolactone were performed in patients with cardiovascular diseases. In 1999, a key clinical trial of spironolactone use in heart failure patients with reduced ejection fraction (≤35%) was published (Randomized ALdactone Evaluation Study (RALES)) [63]. They treated 1663 patients with spironolactone or placebo and the trial was discontinued early because of efficacy. They found a 30% reduction of mortality risk, lower frequency of hospitalizations for worsening heart failure and improvement of heart failure symptoms in the spironolactone group. However, when Pitt et al. used spironolactone and placebo in heart failure patients with preserved ejection fraction (≥45%) (Treatment of Preserved Cardiac Function Heart Failure with an Aldosterone Antagonist (TOPCAT) trial), spironolactone did not reduce the incidence of death from cardiovascular causes, nor did it abort cardiac arrest or reduce hospitalization for the management of heart failure [64]. In 2016, Beygui et al. (Aldosterone Lethal effects Blocked in Acute myocardial infarction Treated with or without Reperfusion to improve Outcome and Survival at Six months follow-up (ALBATROSS) trial) also could not show the advantage of MRA use (one intravenous bolus of potassium canrenoate followed by oral spironolactone for 6 months) added to standard therapy in patients with acute myocardial infarction [65].

#### Spironolactone and DKD

Since spironolactone has the longest history of all the MRA, most of the studies with MRA in DKD were performed with it. Different tissue and animal studies proved its renoprotective effects [66,67,68,69,70,71,72]. In 2001, Miric et al. published a study that showed that short-term treatment with spironolactone in streptozotocin-diabetic rats reversed renal and cardiac fibrosis [66]. In the same animal model, Fujisawa et al. showed that spironolactone attenuated renal fibrosis and suppressed macrophage infiltration, plasminogen activator inhibitor-1 (PAI-1) and transforming growth factor-β1 (TGF-β1) expression in glomeruli and tubulointerstitium [67]. Han et al. showed that spironolactone reduces albuminuria, inflammation and glomerulosclerosis in diabetic rats, and that it inhibits nuclear factor-κB (NF-κB) activation and monocyte chemoattractant protein-1 (MCP-1) production in cultured intrinsic renal cells [68]. Yuan et al. and Pessoa et al. additionally showed that spironolactone reduces oxidative stress in diabetic rats [69,71].

Until today, many studies have been performed on the use of spironolactone in patients with DKD; here, we will highlight just some of them. The first report of proteinuria reduction from spironolactone as an add-on therapy to a RAS inhibitor in chronic kidney disease patients (5 out of 8 had DKD) dates back to 2001 [73]. This was followed by a study by Sato et al. that showed aldosterone escape in 40% of their 45 type 2 DM patients with early nephropathy treated with a RAS inhibitor [74]. These patients had significantly higher albuminuria than patients without aldosterone escape. Adding spironolactone 25 mg to their treatment significantly reduced albuminuria without changes in serum potassium. The first small, double-blinded, controlled, 2-period crossover study was performed by Rosing et al. [75]. Twenty-one type 2 DM patients with nephropathy were treated with spironolactone 25 mg once daily or placebo for 8 weeks, in addition to a maximally recommended dose of a RAS inhibitor. Spironolactone significantly reduced albuminuria and blood pressure, but the change in albuminuria was not correlated with the change in blood pressure. A few months later a similar study in type 1 DM patients was published; they came to the same conclusion as the previous study [76]. The antiproteinuric effect of spironolactone was later also confirmed to be long-lasting (one-year follow-up) in a study of type 2 DM patients with macroalbuminuria on treatment with RAS inhibitors [77]. Takebayashi et al. found that spironolactone inhibits the production of urinary MCP-1 and reduces oxidative stress (urinary 8-iso-prostaglandin F2α) independently of blood pressure in type 2 DM patients with nephropathy [78]. Nielsen et al. found that spironolactone treatment in DKD did not change markers of inflammation and endothelial dysfunction [79]. Later, Mehdi et al. performed a study on 81 patients with DM and arterial hypertension, all receiving lisinopril (80 mg once daily) [80]. They were randomly assigned to receive a placebo, losartan (100 mg daily), or spironolactone (25 mg daily) for 48 weeks. The addition of spironolactone, but not losartan, reduced albuminuria significantly in comparison to placebo. There was no difference in blood pressure changes between groups. In another study, Esteghmati et al. also showed that spironolactone in combination with an ARB is more effective than the combination of an ARB and ACE inhibitor in reducing albuminuria in type 2 DM patients treated for 18 months [81]. Maklough et al. showed that spironolactone alone is as effective as the combination of spironolactone and losartan on albuminuria reduction in type 2 DM patients [82].

Two meta-analyses were published on the treatment of spironolactone in combination with a RAS inhibitor in DKD [83,84]. The addition of spironolactone significantly reduced albuminuria in comparison to monotherapy with a RAS inhibitor [84]. Spironolactone significantly increased the serum/plasma creatinine without a significant change in eGFR [84]. Spironolactone also significantly increased serum/plasma potassium levels and the risk for hyperkalemia [83,84].

The latest research goes in the direction of personalized medicine and optimization of therapy through the identification of urinary metabolites, reflecting inflammation and fibrosis, and consequently predicting albuminuria response of patients with DKD to spironolactone [85]. Mulder et al. identified such a set of 18 urinary metabolites [85]. In the PRIORITY (Proteomic prediction and Renin angiotensin aldosterone system Inhibition prevention of early diabetic nephRopathy In TYpe 2 diabetic patients with normoalbuminuria) study a urinary proteomic risk classifier (CKD273) score was tested in type 2 DM patients with preserved renal function and without albuminuria [86]. They found that CKD273 was associated with an increased risk of progression to albuminuria over a median of 2.5 years, independent of clinical characteristics. However, spironolactone did not prevent progression to albuminuria in these high-risk patients.

### 4.2. Eplerenone

Eplerenone (CGP-30083; 9-11α-epoxymexrenone) is a steroidal MRA, a derivative of spironolactone that has a lower affinity for the MR than spironolactone but a much higher selectivity [45,87]. After binding to the MR it stabilizes it in a transcriptionally inert conformation and does not actively recruit corepressors [88]. It has a higher bioavailability than spironolactone since only fifty percent of it is bound to plasma proteins [60]. Its half-life is 4–6 h and it has no active metabolites [30]. In spite of its short half-life, its anti-mineralocorticoid activity can last up to 12 h, probably because of downstream MR signaling inhibition leading to natriuresis over time [60]. Based on quantitative whole-body autoradiography in rodents it achieves a three times higher concentration in the kidneys than in the heart [60]. Because of its selectivity it lacks the sexual side effects of spironolactone. Hyperkalemia is also an issue in chronic kidney disease patients or with concomitant use of RAS inhibitors [15,89]. First clinical studies with eplerenone date back to 1989, but it was approved by the FDA in 2002 for arterial hypertension and 2003 for symptomatic heart failure with reduced ejection fraction after an acute myocardial infarction [61,90]. It is available in two doses—25 mg and 50 mg. The starting dose for heart failure is 25 mg once daily that can be up titrated to 50 mg once daily, while the starting dose for arterial hypertension is 50 mg once daily and can be increased to 50 mg twice daily [91].

An important study of the effect of eplerenone in arterial hypertension patients was conducted by Weinberger et al. [92]. They showed that eplerenone significantly reduced blood pressure in comparison to placebo without serious side effects. The antihypertensive effect was dose-dependent. However, eplerenone was less effective in blood pressure lowering than spironolactone. Eplerenone 100 mg once daily or 50 mg twice daily had an efficacy of 50–75% compared to spironolactone 50 mg twice daily. A seminal study of eplerenone use was conducted by Pitt et al. [93]. It was a placebo-controlled study in 6632 patients with acute myocardial infarction complicated by left ventricular dysfunction and heart failure (Eplerenone Post-Acute Myocardial Infarction Heart Failure Efficacy and Survival Study (EPHESUS)). During a mean follow-up of 16 months, eplerenone reduced morbidity and mortality in these patients. Another important study in heart failure patients is the Eplerenone in Mild Patients Hospitalization And SurvIval Study in Heart Failure (EMPHASIS-HF) [94]. In this study, 2737 patients with New York Heart Association class II heart failure and an ejection fraction of no more than 35% received eplerenone or placebo, in addition to standard recommended therapy. The study was prematurely stopped since eplerenone significantly reduced the risk of death and hospitalizations for heart failure in comparison to placebo. Included DM patients only had mild hyperkalemia; no significant increase in serum potassium or discontinuation of the drug occurred [95]. Additional analysis of 1846 initially non-diabetic patients from the EMPHASIS-HF study showed that eplerenone had no effect on new-onset DM [96]. Eplerenone was also studied in patients with acute myocardial infarction with ST-elevation without known heart failure, receiving standard medical care [97]. Its use was safe and well tolerated, it reduced the level of brain natriuretic peptides, but no other clear benefit was found.

#### Eplerenone and DKD

Eplerenone was used in animal experiments of DKD. Guo et al. studied uninephrectomized diabetic rats (DM type 1 model) and mice (DM type 2 model) [98]. Diabetic rodents showed increased expression of the MR. In both models, eplerenone significantly reduced albuminuria and kidney lesions (glomerular hypertrophy, mesangial proliferation, tubulointerstitial injury, etc.), and increased renal cortical levels of MR messenger RNA (mRNA), TGF-β mRNA and osteopontin mRNA. These changes were not a result of blood pressure or glycemia changes. Lian et al. studied the effect of eplerenone in a type 1 diabetic rat model [99]. Eplerenone decreased albuminuria, glomerular volume, TGF-β1 expression and glomerular collagen IV without changing glomerular macrophage infiltration. Kang et al. studied the effect of eplerenone and enalapril treatment in type 2 diabetic rats [100]. Both drugs improved albuminuria, glomerular filtration rate and glomerulosclerosis, with the most distinctive changes seen in the combination group. Zhou et al. performed a study in uninephrectomized type 2 diabetic mice [101]. Untreated mice developed progressive albuminuria, glomerulosclerosis, decreased number of podocytes, increased renal expression of fibrotic markers, markers of inflammation and oxidative stress. After treatment with eplerenone, enalapril or both, all these changes were reduced or the progression of changes was arrested, mostly with the combination of both drugs.

Only two published clinical studies exist on the use of eplerenone in type 2 DM patients with albuminuria. The first one was a 24-week, double-blind study of 215 type 2 DM patients with mild-to-moderate arterial hypertension and albuminuria (UACR ≥50 mg/g) [102]. Patients randomly received eplerenone 200 mg or enalapril 40 mg or the combination of eplerenone 200 mg and enalapril 10 mg once daily. Eplerenone alone or in combination caused a significant reduction of UACR, independent of blood pressure reduction. Hyperkalemia was an issue; therefore, another study was conducted with lower eplerenone doses. In this study, 268 type 2 DM patients with albuminuria (UACR ≥ 50 mg/g) after an open-label run-in with enalapril 20 mg daily were assigned to a 12-week treatment with eplerenone 50 mg or 100 mg or placebo once daily [103]. UACR was significantly reduced from baseline in both eplerenone groups (50 mg: 41.0%, 100 mg: 48.4%) but not in the placebo group (7.4%). No significant differences in hyperkalemia between groups were detected. Even so, according to the label, eplerenone prescription is contraindicated in patients with renal dysfunction or for the treatment of arterial hypertension in type 2 DM patients with albuminuria [91].

## 5. Non-Steroidal MRA

### 5.1. Apararenone

Apararenone (MT-3995; *N*-[4-(4-fluorophenyl)-2,2-dimethyl-3-oxo-3,4-dihydro-2*H*-1,4-benzoxazin-7-yl]methanesulfonamide) is a benzoxazinone derivative [104]. It is a long-acting, highly selective MRA [105,106]. Its major metabolite is 1118174, which has 1/50 of apararenone’s affinity to bind to the MR. Currently, very little data are available about apararenone. It was used in a phase 2 clinical trial in patients with nonalcoholic steatohepatitis, but data are not yet published [87].

The only published study of apararenone use was performed in DKD patients [107]. It included 293 Japanese patients with type 2 DM and albuminuria. In the first part of the study, patients received placebo or different dosages of apararenone for 24 weeks. After this, some of the patients were included in an uncontrolled extension study of 28 weeks. Apararenone significantly reduced UACR in comparison to placebo. After 24 weeks, the percent reduction from baseline in UACR was around 40–50% in all patients receiving apararenone. The UACR lowering effect was confirmed with or without concomitant use of RAS inhibitors. After 52 weeks, the percent reduction from baseline in UACR was approximately 60% in apararenone 5 mg and 10 mg groups. eGFR decreased, while serum potassium increased, but changes were not clinically significant. In this study, apararenone appeared to be safe and tolerable; an appropriate clinical dose would probably be 5 mg or 10 mg once daily. 

### 5.2. Esaxerenone

Esaxerenone (CS-3150; (5*S*)-1-(2-hydroxyethyl)-4-methyl-*N*-[4-(methylsulfonyl)phenyl]-5-[2-(trifluoromethyl)phenyl]-1*H*-pyrrole-3-carboxamide) is a selective and highly potent MRA [87]. Its structure is based on the dihydropyridine calcium antagonist. It is rapidly absorbed, has a long elimination half-life (around 20–30 h) and a dose proportional exposure [108,109]. Quantitative whole-body autoradioluminography in rats showed that it is widely distributed to the whole body, with a similar distribution to the heart and the kidneys and with a low distribution to the central nervous system [104,110]. Serious adverse events are rare, with the most common being hyperkalemia [111]. Moderate renal impairment (eGFR 30–60 mL/min/1.73 m^2^) and mild or moderate hepatic impairment (Child-Pugh A or B) do not alter esaxerenone’s pharmacokinetics in a clinically significant way. However, the effect of severe cases of renal or hepatic impairment on pharmacokinetics is still unknown [111]. Esaxerenone has been mostly investigated in Japan and in 2019 it there received marketing approval as an antihypertensive agent [111]. It is available as 1.25 mg, 2.5 mg and 5 mg tablets, with the recommended dosage being 2.5 mg once daily. If this is insufficient, the dosage can be increased to 5 mg.

Before approval as an antihypertensive drug, two important clinical trials were performed. The first one was a phase 2, multicenter, randomized, double-blind, placebo-controlled, open-label comparator study in Japanese patients with arterial hypertension [112]. After 12 weeks of treatment, esaxerenone was shown to be an effective and tolerable treatment. The same proved to be true in a similar phase 3 study and later also in hypertensive patients with moderate kidney dysfunction [113,114]. Until now, esaxerenone has not been studied for other indications, except DKD.

#### Esaxerenone and DKD

In kidney disease, esaxerenone first showed promise by reducing proteinuria and kidney injury in rats [115,116,117]. Bhuyian et al. were the first to study the effect of esaxerenone in mice with DKD [118]. They found that esaxerenone or spironolactone reduced blood pressure to a similar degree, but esaxerenone caused a greater decrease of albuminuria, glomerular injury, tubulointerstitial fibrosis and renal inflammation than spironolactone. This was associated with a decrease in renal oxidative stress. In another study on diabetic mice, Arai et al. found that the renoprotective effects of esaxerenone could be independent of its antihypertensive effect [119].

A study in Japanese patients with arterial hypertension, type 2 DM and albuminuria (UACR 30–999 mg/g) was performed by Itoh et al. [120]. Fifty-one patients received esaxerenone for 12 weeks in a gradually higher dose as an add-on therapy to a RAS inhibitor. Esaxerenone had an additional antihypertensive effect and it significantly reduced albuminuria. The risk of hyperkalemia was made low by adjusting the dose to the patient’s serum potassium level, kidney function and blood pressure. A phase 2b trial in Japanese patients with type 2 DM and albuminuria was also performed by Ito et al. [121]. It was a multicenter, randomized, double-blind, placebo-controlled trial. They included 365 hypertensive or normotensive patients with type 2 DM and albuminuria (UACR ≥ 45 to < 300 mg/g). All patients had an eGFR ≥ 30 mL/min/1.73 m^2^ and were treated with a RAS inhibitor. They received esaxerenone or placebo for 12 weeks. Esaxerenone treatment significantly reduced UACR (38–56%) in comparison to placebo. The most common adverse event was hyperkalemia, which was dose-proportional. Another phase 3 trial in Japanese type 2 DM patients with albuminuria (Esaxerenone in Diabetic Nephropathy (ESAX-DN) study) followed [122]. In total, 455 patients received esaxerenone or placebo for 52 weeks. Esaxerenone was associated with a > 30% reduction in UACR in about 70% of patients. UACR returned to < 30 mg/g in 22% of patients and there was a 76% reduction in hazard of transition to UACR ≥ 300 mg/g. Compared to placebo, more patients in the esaxerenone group had a serum potassium level ≥ 6.0 or ≥ 5.5 mEq/L on two successive measurements. Ten (4%) patients in the esaxerenone and one (1%) in the placebo group stopped treatment because of hyperkalemia. Hyperkalemia was asymptomatic and resolved after dosage reduction or treatment discontinuation.

### 5.3. Finerenone

Finerenone (BAY 94-8862; (4*S*)-4-(4-cyano-2-methoxyphenyl)-5-ethoxy-2,8-dimethyl-1,4-dihydro-1,6-naphthyridine-3-carboxamide) is a potent, highly selective, full antagonist of the MR [60]. In contrast with spironolactone and eplerenone, which bind to the ligand domain of the MR, finerenone induces a conformational change within the MR complex, thereby changing the stability and nuclear translocation of the receptor [14]. Structurally, it is a dihydropyridine derivative but it does not have pronounced activity at the L-type calcium channel [61]. It has a half-life of 2 h and no active metabolites [60]. Finerenone is less lipophilic and has greater polarity than steroidal MRA [88]. Therefore, it is less capable of traversing the blood–brain barrier and interfering with MR in the central nervous system [15]. These qualities are believed to have a significant role in the control of blood pressure [88]. Based on quantitative whole-body autoradiography in rodents, it reaches the same concentration in the kidneys and the heart [60]. Finerenone can also cause hyperkalemia, but according to current studies, it occurs in a significantly lower proportion than steroidal MRA [123]. Finerenone has been used in clinical research in doses from 1.25 mg to 20 mg once daily, although in the latest two clinical trials they used 10 mg or 20 mg once daily depending on eGFR.

Animal studies showed that finerenone has anti-inflammatory and anti-fibrotic effects and consequently beneficial cardiorenal effects [124,125,126,127]. Therefore, clinical trials followed. The minerAlocorticoid Receptor antagonist Tolerability Study (ARTS) was a phase 2a clinical trial that assessed the safety and tolerability of finerenone in patients with heart failure with reduced ejection fraction and mild or moderate chronic kidney disease [128]. Finerenone 5–10 mg/day was at least as effective as spironolactone 25 mg or 50 mg/day in decreasing biomarkers of hemodynamic stress with lower incidences of hyperkalemia and worsening renal function. A phase 2b, randomized, double-blind trial, minerAlocorticoid Receptor antagonist Tolerability Study-Heart Failure (ARTS-HF) followed [129]. In this study, 1066 patients with type 2 DM and/or chronic kidney disease, who presented in emergency departments with worsening chronic heart failure with reduced ejection fraction were randomized. They received oral, once-daily finerenone or eplerenone for 90 days. Finerenone was well tolerated and induced a 30% or greater decrease in N-terminal pro-brain natriuretic peptide (NT-proBNP) levels in a similar proportion of patients to eplerenone. The composite endpoint of death from any cause, cardiovascular hospitalization or emergency presentation for worsening heart failure occurred less frequently with finerenone compared to eplerenone, also with a smaller mean increase in serum potassium [130].

#### Finerenone and DKD

The minerAlocorticoid Receptor antagonist Tolerability Study-Diabetic Nephropathy (ARTS-DN) was a randomized, double-blind, placebo-controlled, phase 2b study on 823 type 2 DM patients with albuminuria (UACR ≥ 30 mg/g), an eGFR higher than 30 mL/min/1.73 m^2^, being treated with at least the minimum recommended dosage of a RAS inhibitor before the screening visit and with a serum potassium concentration ≤ 4.8 mmol/L [131]. They received different doses of oral finerenone once daily or placebo for 90 days. Finerenone demonstrated a dose-dependent reduction in UACR. Hyperkalemia and subsequent discontinuation of finerenone occurred in 1.8%, compared with no cases in the placebo group. There were no differences in the incidence of eGFR decrease of ≥30% or incidences of adverse and serious adverse events between the placebo and finerenone groups. Additionally, finerenone did not affect glycated hemoglobin (HbA1c) levels [130]. Since the abovementioned study was of shorter duration and since UACR is not a surrogate marker of renal outcome, an upgraded study, FIDELIO-DKD (FInerenone in reducing kiDnEy faiLure and dIsease prOgression in Diabetic Kidney Disease), followed [132]. This was a phase 3 study that included 5734 patients with chronic kidney disease and type 2 DM and followed them for a median of 2.6 years. Chronic kidney disease was defined according to one of two sets of criteria: UACR 30–300 mg/g, eGFR 25–60 mL/min/1.73 m^2^ and a history of diabetic retinopathy or UACR 300–5000 mg/g and eGFR 25–75 mL/min/1.73 m^2^. All patients were treated with a RAS inhibitor at the maximum dose on the manufacturer’s label that did not cause unacceptable side effects and they were required to have a serum potassium level ≤4.8 mmol/L. In the study they found that finerenone lowered the risk of primary (kidney failure, a sustained decrease of ≥40% in the eGFR from baseline or death from renal causes) and secondary outcome events (death from cardiovascular causes, nonfatal myocardial infarction, nonfatal stroke or hospitalization for heart failure) in comparison to placebo. Hyperkalemia was more common in the finerenone group, but discontinuation of the trial due to it was uncommon (2.3%). An additional subgroup analysis of these data showed that finerenone lowered the incidence of the composite cardiovascular outcome independent of preexisting cardiovascular diseases [133]. Another big phase 3 clinical trial was FIGARO-DKD (FInerenone in reducinG cArdiovascular moRtality and mOrbidity in Diabetic Kidney Disease), that investigated the role of finerenone in reducing major cardiovascular events, with slowing DKD progression as a prespecified secondary endpoint [15,134]. It was a randomized, double-blind, placebo-controlled trial. They randomized 7437 patients with an eGFR ≥ 25 mL/min/1.73 m^2^ and UACR 30–5000 mg/g to therapy with finerenone or placebo. The primary endpoint was the composite of time to cardiovascular death or non-fatal cardiovascular event (myocardial infarction, stroke or hospitalization for heart failure). A main secondary endpoint was the composite of time to kidney failure, a sustained reduction of eGFR of ≥40% or renal death. Other secondary endpoints were time to all-cause mortality, all-cause hospitalization, UACR change from baseline to month 4 and a composite endpoint of time to first occurrence of kidney failure or sustained decrease of eGFR ≥57% from baseline over at least 4 weeks or renal death. The trial was completed, but the results are not yet published. In May 2021, only a special press release announced that the primary endpoint of the study was reached [135]. 

The latest meta-analysis, including randomized, controlled trials on the addition of MRA to RAS inhibitors in DKD, was published in 2019 [84]. The authors performed a subgroup analysis that suggested a lower relative risk of hyperkalemia with finerenone than with eplerenone or spironolactone. However, a limitation is that for finerenone only the first published study (ARTS-DN) was used for this analysis. 

## 6. Conclusions

MRA have a role in DKD in lowering albuminuria. Hard outcomes such as kidney disease progression, cardiovascular events and mortality data are missing for all MRA except finerenone. The use of steroidal MRA in DKD has been limited by its side effects, especially hyperkalemia. Perhaps with sufficient attention to diet, ACE inhibitor/ARB use, potassium binder use, kidney function, regular serum potassium controls and thorough dose adjustment their use could be more widespread. With the advent of non-steroidal MRA that cause less hyperkalemia, their use in DKD might even become everyday clinical practice. Table 1 summarizes an overview of MRA used in everyday clinical practice and the latest clinical research. MRA still represent an exciting and growing research field. The goal is to find a selective antagonist with a tissue and function-specific mode of action. In the future, coregulators of MR may represent the next generation of MR modulators. 

## Figures and Tables

**Table 1:** Overview of mineralocorticoid receptor antagonists (MRA) used in everyday clinical practice and latest clinical research.

	Spironolactone (SC-9420)	Eplerenone (CGP-30083)	Apararenone (MT-3995)	Esaxerenone (CS-3150)	Finerenone (BAY 94-8862)
**Type of MRA**	Steroidal	Steroidal	Non-steroidal (benzoxazinone derivative)	Non-steroidal (dihydropyridine derivative)	Non-steroidal (dihydropyridine derivative)
**Potency**	++	+	+	+++	+++
**Selectivity**	+	++	+++	+++	+++
**Half-life**	1–2 h	4–6 h	Long (approximately 250–300 h)	20–30 h	2 h
**Major metabolite(s)**	7α-thiomethyl-spironolactone Canrenone (half-life: 18–24 h)	None	1118174	M4, M11, M1	None
Tissue distribution *	6× higher concentration in the kidneys than in the heart	3× higher concentration in the kidneys than in the heart	Unknown	Same concentration in the kidneys and the heart, low concentration in the CNS	Same concentration in the kidneys and the heart
**Approved for use**	Edema AH Primary hyperaldosteronism	AH Symptomatic HFrEF after AMI	/	AH (Japan)	/
**Dosing**	Ascites due to cirrhosis: 100–400 mg/day Symptomatic HFrEF: 12.5–50 mg/day AH: 25–100 mg/day Primary hyperaldosteronism: 12.5–400 mg/day	AH: 50 mg 1–2×/day Symptomatic HFrEF after AMI: 25–50 mg/day	In research: 5 mg or 10 mg/day	2.5–5 mg/day	In research: 10 mg or 20 mg/day
**Side effects**	Hormonal (gynecomastia, impotence, menstrual irregularities) Hyperkalemia	Hyperkalemia	Unknown	Hyperkalemia	Hyperkalemia
**Important clinical studies in cardiovascular medicine**	RALES study [63] TOPCAT trial [64] ALBATROSS trial [65]	Weinberger et al. [92] EPHESUS [93] EMPHASIS-HF [94] Montalescot et al. [97]	None	Ito et al. [112] Ito et al. [113]	ARTS [128] ARTS-HF [129]
**Important clinical studies in diabetic kidney disease**	Rossing et al. [75] Schojedt et al. [76] van den Mairacker et al. [77] Mehdi et al. [80] Hou et al. [83] Zou et al. [84]	Epstein et al. [102] Epstein et al. [103]	Wada et al. [107]	Itoh et al. [120] Ito et al. [121] ESAX-DN [122]	ARTS-DN [131] FIDELIO-DKD [132] FIGARO-DKD [134]

Legend: * Based on quantitative whole-body autoradiography in rodents; AH—arterial hypertension, AMI—acute myocardial infarction, CNS—central nervous system, HFrEF—heart failure with reduced ejection fraction, / = not approved for use.

## Data Availability

No new data were created or analyzed in this study. Data sharing is not applicable to this article.
